# Supplementary material for: Theory-based immunisation health education intervention in improving child immunisation uptake among antenatal mothers attending federal medical centre in Nigeria: A study protocol for a randomized controlled trial
Source: PLoS One. 2022 Dec 8;17(12):e0263436. doi: 10.1371/journal.pone.0263436 (PMC9731461; doi:10.1371/journal.pone.0263436)
Supplement: S1 File — (DOCX) [file pone.0263436.s002.docx]

## 3.6 Sample Size Determination

The minimum sample size required to test the proposed study hypotheses has been determined using the formula for comparing two proportions in selected samples of both intervention and control groups (Lwanga & Lemeshow, 1991). This formula gave the required number (n) for each group.

n = [Z1-α/2 √ (2P̄ (1-P̄)) +Z1-β √P1(1-P1) +P2(1–P2)]2

(P1- P2)2

In order to calculate the sample size, many parameters from different studies were substituted into the formula. Nevertheless, the parameters from multi-site community-based intervention study for mother-infant pairs in Karachi, Pakistan (Owais, Hanif, Siddiqui, Agha & Zaidi, 2011) is adopted due to the fact that they provide the highest sample size compared with (Oche, Umar, Ibrahim & Sabitu, 2011) thus:

P1= proportion of subjects in the intervention group receiving all 3 doses of DPT/Hepatitis B three months after the baseline survey (0.721).

P2 = proportion of subjects in the control group receiving all 3 doses of DPT/Hepatitis B three months after the baseline survey (0.517).

P̄ = average proportion of all subjects receiving all 3 doses of DPT/Hepatitis B three months after the baseline survey ((P1 + P2)/2) =

(0.721+0.517)

2

With Z1-α/2 of 1.96 and Z1-β of 80% power = 0.84, this provided a minimum sample size (n_0_) of 88 participants in each group.

Adjustments were then made for estimated response rate and estimated eligibility (Aday & Cornelius, 2006) thus:

1. Adjustment for the estimated response rate, where the estimated attrition rate (L) is 50%

n1 = n_0_ /(1-L) = 88 / (1-0.5) = 176

1. Adjustment for estimated eligibility (90%)

n2 = n1 / 0.9 = 176 / 0.9 =196

This provided a total sample size of 196 participants in each group, which will then be multiplied by two since the study is comprised of two arms. Therefore, 392 are the calculated sample size required to test the hypothesis for this study.
