# Supplementary material for: Theory-based immunisation health education intervention in improving child immunisation uptake among antenatal mothers attending federal medical centre in Nigeria: A study protocol for a randomized controlled trial
Source: PLoS One. 2022 Dec 8;17(12):e0263436. doi: 10.1371/journal.pone.0263436 (PMC9731461; doi:10.1371/journal.pone.0263436)
Supplement: S2 File — (DOCX) [file pone.0263436.s003.docx]

##### Table 2. Tabular illustration of the immunization intervention modules by constructs and contents

| Theory Construct | Health education Contents | Module & strategy | Time Duration |
| --- | --- | --- | --- |
| Knowledge | - Introduction to immunization - Primary objective of child immunization. - Information on Nigeria childhood immunization guideline - National program on immunization (NPI) child immunization schedule. - Type of vaccines and time to be given - Types of vaccine side effect and their management. - Vaccine preventable diseases and their types. | Module1& module2 (lectures) | 30 minutes and 30 minutes |
| Outcome expectation | - Benefit of childhood immunization uptake and correct their misconception - Consequences of not vaccinating a child: severity and complications of Vaccine Preventable Diseases (VPDs). | Module3 (Lectures and demonstration) | 30 minutes |
| Attitude  Environment  (Social norms) | - Participant experience and those of other mothers - Participant will be encouraged to: receive Maternal Toxoid (TT) vaccine, deliver at hospital and attend their post-natal care follow-up - Brief lectures from traditional and religious leaders with regards to vaccine safety and acceptability to improve cultural and religion beliefs | Module 4 (Interactive discussion, brain storming, videos and lectures) | 1 hour |
| Problem solving | - Sharing session on the challenges and how to solve it. | Module 4 (Interactive section and brain storming) | 30 minutes |
| Self-efficacy | - Empowering mothers with knowledge to comply with childhood immunization uptake (verbal persuasion) - Evaluation of self-efficacy for complying with childhood immunization uptake | Module 5 (interactive discussion, brain storming and role play) | 1 hour |
| Goal settings | - Antenatal mothers will be informed about the compliance of child immunization schedule and encouraged them to set the goal | Module 5 (Lectures and interactive discussion) | 30 minutes |
| Reinforcement | - Mothers will be receiving a mobile call reminder at 7 and 1 days before their childhood immunization schedule | Mobile reminder |  |
