# Supplementary material for: Theory-based immunisation health education intervention in improving child immunisation uptake among antenatal mothers attending federal medical centre in Nigeria: A study protocol for a randomized controlled trial
Source: PLoS One. 2022 Dec 8;17(12):e0263436. doi: 10.1371/journal.pone.0263436 (PMC9731461; doi:10.1371/journal.pone.0263436)
Supplement: S3 File — (DOCX) [file pone.0263436.s004.docx]

## Operational Definitions

Below are the operational definitions of the respondents (table 3.2):

**Table 3.2 Operational Definitions Table**

| No. | Variables | Definition |
| --- | --- | --- |
| Section A | Sociodemographic of respondent | This refers to the sociodemographic of respondent’s which include: maternal age, marital status, no children, maternal educational status, paternal educational status, maternal occupation, religion, ethnicity, monthly income and place of resident. |
|  | Maternal age | This refers to the age of mother at the time of delivery which is been categorized into two different groups; below 19 or above 19 years of age (Negussie, Kassahun, Assegid & Hagan, 2015). |
|  | Marital status | This refers to the respondent’s official marital status which is been categorized into five different groups single, married, divorced or widowed (Anokye et al., 2018). |
|  | No of children | This refers to the numbers of children having per mother (Oliveira, Martinez & Rocha, 2014). |
|  | Maternal educational status | This refers to educational status of mother which could be no-formal education, primary school education, secondary school education or tertiary education (Kiptoo, Esilaba, Kobia & Ngure, 2015). |
|  | Paternal educational status | This refers to educational status of father which could be no-formal education, primary school education, secondary school education or tertiary education (Legesse & Dechasa, 2015). |
|  | Maternal occupation | This refers to occupational status of mothers and it is been classified into four categories; house wife, student, trader, civil servant or farmer (Legesse & Dechasa, 2015). |
|  | Religion | The respondent’s religion status for this study is categorized into Muslim and non-Muslim. |
|  | Ethnicity | The respondent’s ethnicity for this study is categorized into Hausa and non-Hausa |
|  | Monthly income | This refers to the amount of income a family earn in a month and in this study, it is categorized into less than 18000 or above 18000 naira (Nigerian currency). |
|  | Place of resident | Area of residence was described as a place where people are living which could either be a rural or urban area **(**Kiptoo, 2015). |
| Section B | Obstetric history of respondent | This refers to the number of times attended ANC follow-up (Legesse & Dechasa, 2015), birth interval between last born and current pregnancy (Rahman & Obaida-Nasrin, 2010) and whether or not mothers receive any Tetanus toxoid vaccine (Adedire et al., 2016). In this study, number of times attended ANC follow-up is categorized into either less than three times or more-than three times. Birth interval between last born and current pregnancy was classified into three categories: first pregnancy, less than 48 months or more than 48 months. For whether or not mothers receive any Tetanus toxoid vaccine, it was classified as either yes or no. |
| Section C | Health care system of respondent | This refers to health care infrastructure or resourcing which includes: distance to health facility (Legesse & Dechasa, 2015), attitude of the hospital staff (Chambongo, Nguku, Wasswa & Semali, 2016), accessibility of vaccine site (Animaw et al, 2014) and mode of transportation to reach hospital (Odutola et al., 2015). In this study, distance to health facility is classified into either less than 30 minutes or more than 30 minutes, attitude of hospital staff could either be good or poor, hospital accessibility either yes or no and means of transportation to hospital either car/motorcycle or walking. |
| Section D | Knowledge | This refers to the knowledge of respondent’s regarding general knowledge on childhood immunization, knowledge on child immunization schedule, knowledge on vaccine side effects and management and knowledge on sign, symptoms and mode of transmission of VPDs. |
| Section E | Attitudes | This refers to the attitudes of respondent’s towards childhood immunization uptake. |
| Section F | Outcome expectation | This refers to the outcome expectation of respondent’s towards childhood immunization uptake (important of vaccinating a child and consequences that may arise for non-compliance) |
| Section G | Cultural beliefs | This refers to the cultural beliefs of respondent’s towards childhood immunization uptake. |
| Section H | Assumption on religious regulations | This refers to the assumption on religious regulations of respondent’s towards childhood immunization uptake. |
| Section I  Section J  Section K  Section L  Section M  Section N  Section O  Section P | Self-efficacy  Knowledge score  Attitude score  Outcome expectation score  Cultural belief score  Assumption on religious regulations score  Self-efficacy scores  Childhood immunization uptake | This refers to the self-efficacy of respondent’s towards childhood immunization uptake.  This referred to the total scores of knowledge for all items of the knowledge section as answer by the respondent.    In our analysis, this referred to the total scores of attitudes for all items under attitude section as answer by the respondent.  In our analysis, this referred to the total scores of outcome expectation for all items under outcome expectation section as answer by the respondent.  In our analysis, this referred to the total scores of cultural beliefs for all items under cultural belief section as answer by the respondent.  In our analysis, this referred to the total scores of assumptions on religious regulations for all items under assumptions on religious regulations section as answer by the respondent.  In our analysis, this referred to the total scores of self-efficacies for all items under self-efficacy section as answer by the respondent.  In our analysis, a child will be considered to be fully immunized if he/she received one dose of BCG vaccine, all the four doses of OPV and three doses of pentavalent and pneumococcal conjugate vaccine latest by the age of six months with an intervals of at least four weeks between first and subsequent doses as recommended by WHO (assessed through child vaccination cards or hospital record). A child will be considered to be non-immunized if he/she fail to receive any dose for OPV, PCV, BCG or Pentavalent vaccine (WHO, 2019c). |
