# Supplementary material for: Theory-based immunisation health education intervention in improving child immunisation uptake among antenatal mothers attending federal medical centre in Nigeria: A study protocol for a randomized controlled trial
Source: PLoS One. 2022 Dec 8;17(12):e0263436. doi: 10.1371/journal.pone.0263436 (PMC9731461; doi:10.1371/journal.pone.0263436)
Supplement: S4 File — (DOC) [file pone.0263436.s005.doc]

**10/11/2020 Version 2**

**
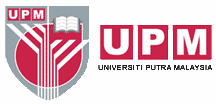
 FORM 2.4: RESPONDENT’S INFORMATION SHEET AND INFORMED CONSENT FORM**

**JAWATANKUASA ETIKA UNIVERSITI UNTUK PENYELIDIKAN MELIBATKAN MANUSIA (JKEUPM)**

**UNIVERSITI PUTRA MALAYSIA, 43400 UPM SERDANG,**

**SELANGOR, MALAYSIA**

Please read the following information carefully and do not hesitate to discuss any questions you may have with the researcher.

1. **STUDY TITLE :**

Effect of Immunization Health Education Intervention in Improving Child Immunization Uptake Among Antenatal Mothers Attending Federal Medical Center in Nigeria.

1. **INTRODUCTION:**

The World Health Organisation (WHO) has described immunization as the process through which the immunity or resistance of individual to infectious diseases is built. Despite the efforts made by health organizations to combat disease, vaccine preventable diseases have remained the most common cause of childhood mortality, which is estimated at 3 million deaths around the world annually. From a report done by WHO and United Nations International Children’s Emergency Fund (UNICEF) it was revealed that 2.5 million deaths occurred every year due to vaccine preventable diseases, mainly in Africa and Asia among children less than 5 years old. In Nigeria, Vaccine Preventable Diseases accounted for 17 and 22 under-five childhood morbidity and mortality respectively. unlike developed country like England where child immunization coverage was 93.6% and developing country like Malaysia ≥95%, in Nigeria, routine vaccination coverage for all recommended vaccines has remained poor. However, there was an increase in vaccination coverage from 25.4% of eligible children (12–23 months of age) in 2013 to 31% in 2018. There is wide variation with in the geopolitical zones and state concerning the full vaccination coverage of children; 52% in the southeast and south-south zones and 10% in the northwest. In Zamfara state, only 7.4% of the children are fully immunized. Lack of adequate knowledge, attitude, outcome expectation, self-efficacy, cultural beliefs and assumptions on religious regulation were the major reasons contributing to poor childhood immunization uptake in Nigeria especially the northwestern part of the country. This study will be conducted to evaluate the effect of immunization health education intervention on knowledge, attitude, outcome expectation, self-efficacy, cultural and assumptions on religious regulation of pregnant women towards childhood immunization uptake in FMC Gusau, Zamfara State, Nigeria.

1. **WHAT WILL YOU HAVE TO DO?**

Your engagement in this study is voluntary. You have the authority to withdraw from this research at any time without mentioning any reason for withdrwaing. If you agree to participate in this study, you will be expected to fill up the questionnaire on your socio-demographic status, obstetric history, health care system and answer the questions (statements) relating to your level of knowledge, attitude, outcome expectation, self-efficacy, cultural beliefs and assumptions on religious regulation regarding childhood immunization uptake.

**10/11/2020 Version 2**

**4. WHO SHOULD NOT PARTICIPATE IN THE STUDY?**

Mothers without good understanding of Hausa language as the intervention will be delivered in Hausa language, mothers who attended immunization health education intervention previously before our study and Mothers of family with nomadic lifestyle.

5. **WHAT WILL BE THE BENEFITS OF THE STUDY:**

**(a)TO YOU AS THE SUBJECT?**

This is will be an important opportunity to improve your knolwdege, attitude, outcome expectation, self-efficacy, cultural beliefs and assumptions on religious regulation towards childhood immunization uptake which will enhance your childhood immunization uptake and therefore preventing your child from having vaccine preventable diseases in future.

**(b)** **TO THE INVESTIGATOR?**

The findings from the study will contribute towards improving knowledge, attitude, outcome expectation, self-efficacy, cultural beliefs and assumptions on religious regulation of antenatal mothers regarding childhood immunization uptake which will increase childhood immunization uptake and decrease mortality and morbidity among children in Nigeria. The intervention module developed from the study may be adopted and consolidated into the routine health education delivering to pregnant women at the time of their antenatal care visits. This will provide better knowledge to antenatal mothers concerning the important of childhood immunization. The study findings could benefit policy makers in making decisions and formulating appropriate guideline and policies with regards to childhood immunization uptake in order to reduce childhood mortality and morbidity of VPDs.

**6. WHAT ARE THE POSSIBLE RISKS?**

This research does not have any direct risk other than perhaps comsuming time.

7. **WILL** **THE INFORMATION THAT YOU PROVIDE AND YOUR IDENTITY REMAIN CONFIDENTIAL?**

Yes the information you will provide will be confidential and solely for the purpose of research.

**8. WHO SHOULD YOU CONTACT IF YOU HAVE ADDITIONAL QUESTIONS DURING THE COURSE OF THE RESEARCH?**

If you have questions related to this study please do not hesitate to contact

1. Abubakar Nasiru Galadima GS52606

PhD student, Public Health

Department of Community Health

Faculty of Medicine and Health Science

Email: [galadimamafara@gmail.com](mailto:galadimamafara@gmail.com)

1. Assoc Prof Dr Nor Afiah Mohd Zulkefli

Head of Department/Main Supervisor

Department of Community Health

Faculty of Medicine and Health Science

Email: [norafiah@upm.edu.my](mailto:norafiah@upm.edu.my)

*Please initial here if you have read and understood the contents of this page______*

**9. CONSENT**

I …………………………………… Identity Card No. …………………………… address………………………………………………………………………………………………………... ……………………………………………………..hereby voluntarily agree to take part in the research stated above *(clinical /drug trial/video recording/ focus group/interview-based/ questionnaire-based).

I have been informed about the nature of the research in terms of methodology, possible adverse

effects and complications (as written in the Respondent’s Information Sheet). I understand that I have the right to withdraw from this research at any time without giving any reason whatsoever. I also understand that this study is confidential and all information provided with regard to my identity will remain private and confidential.

I* wish / do not wish to know the results related to my participation in the research

I agree/do not agree that the images/photos/video recordings/voice recordings related to me be used in any form of publication or presentation (if applicable)

* delete where necessary

Signature ……..………………………… Signature ……..………………………….

(Respondent) (Witness)

Date :………………………………….….. Name :………………………………….…..

I/C No. :………………………………….…..

I confirm that I have explained to the respondent the nature and purpose of the above-mentioned research.

Date ……..………………………… Signature ……..………………………….

(Researcher)
