# Supplementary material for: Theory-based immunisation health education intervention in improving child immunisation uptake among antenatal mothers attending federal medical centre in Nigeria: A study protocol for a randomized controlled trial
Source: PLoS One. 2022 Dec 8;17(12):e0263436. doi: 10.1371/journal.pone.0263436 (PMC9731461; doi:10.1371/journal.pone.0263436)
Supplement: S5 File — (DOCX) [file pone.0263436.s006.docx]

# Section A: Sociodemographic characteristics

**Instruction:** Please tick (√) on the appropriate answer and specify where necessary.

1. Maternal age……………. years old
2. Marital status
3. Single
4. Married
5. Divorced
6. Widowed
7. No of children you are having……….
8. Maternal educational status
9. No education
10. Primary education
11. Secondary education
12. Tertiary education
13. Paternal educational status
14. No education
15. Primary education
16. Secondary education
17. Tertiary education
18. Maternal occupation
19. House wife
20. Student
21. Trader
22. Civil servant
23. Farmer
24. Religion
25. Muslim
26. Christian
27. Others
28. Ethnicity
29. Hausa
30. Non-Hausa
31. Monthly income…………………Naira
32. Place of resident
33. Urban
34. Rural

# Section B: Obstetric history

**Instruction:** Please tick (√) on the appropriate answer and specify where necessary.

1. How many times do you went for antenatal follow-up?
2. < 3 times
3. > 3 times
4. What are the intervals between this pregnancy and your last born?
5. No interval (this is my first pregnancy)
6. < 48 months
7. > 48 months
8. Did you receive any Tetanus Toxoid (TT) vaccine during this pregnancy?
9. Yes
10. No

# Section C: Health care system

**Instruction:** Please tick (√) on the appropriate answer and specify where necessary.

1. How long does it take you to reach this hospital? ……………(minutes)
2. How can you relate the attitude of the hospital staff towards your childhood immunization?
3. Poor
4. Good

1. Is the hospital accessible for you to obtain your child immunization uptake?
2. Yes
3. No
4. What means of transportation are you using to reach the hospital?
5. Private car
6. Private motorcycle
7. Public car
8. Public motorcycle
9. Walking
10. Others …………………

# Section D: knowledge

**Instruction:** Kindly fill in the box by (√) the options that best represent your perspective for the statement/options as follow:

1. Yes
2. No
3. Don’t know

|  | **Statements** | **Yes** | **No** | **Don’t know** |
| --- | --- | --- | --- | --- |
|  | Immunization refers to the process where by an individual’s immune system provide protection against an infectious agent. |  |  |  |
|  | The National Program on Immunization (NPI) ensures every child receive immunization against Vaccine Preventable Diseases (VPDs) before reaching 1 year of age. |  |  |  |
|  | There is a specific type of vaccine for each disease. |  |  |  |
|  | There is specific period for vaccination. |  |  |  |
|  | The first immunization is immediately after birth. |  |  |  |
|  | Vaccination prevents certain infectious diseases |  |  |  |
|  | Vaccination enhance immunity of the child |  |  |  |
|  | Completion of child vaccination as per schedule is important in order to receive good protection against vaccine preventable diseases. |  |  |  |
|  | Pentavalent vaccine, oral polio vaccine, measles vaccine and yellow fever vaccine are provided by government. |  |  |  |
|  | It is very important for me to keep my child immunization record in order for him/her to be fully immunized. |  |  |  |
|  | Bacillus Calmette Guerin (BCG), Oral Polio Vaccine-0 and Hepatitis B-1 are the vaccines given to babies immediately after birth. |  |  |  |
|  | There are some vaccines given to babies at 6-weeks of age, 10 weeks of age and 14 weeks of age. |  |  |  |
|  | According to National Program on Immunization, every child must receive 1 dose of BCG vaccine, 4 doses of OPV, 1 dose of Hep B vaccine, 3 doses of pentavalent vaccine and 3 doses of PCV, 2 doses of measles vaccine and 1 dose of yellow fever vaccine before reaching 1 year of age. |  |  |  |
|  | Fever and a small sore at the site of injection are the general side effects of vaccine. |  |  |  |
|  | In order to manage the small sore, it must be kept dry and clean without applying any ointment or medicine on it. |  |  |  |
|  | Paracetamol syrup and a tepid sponging can be used to manage vaccine side effect. |  |  |  |
|  | A vaccine-preventable disease (VPDs) is an [infectious disease](https://en.wikipedia.org/wiki/Infectious_disease) caused by microbes, of which has an effective preventive [vaccine](https://en.wikipedia.org/wiki/Vaccine). |  |  |  |
|  | Poliomyelitis, tuberculosis, hepatitis B infection, measles and yellow are examples of vaccine preventable diseases. |  |  |  |
|  | Pain or stiffness of arm or leg are symptoms of vaccine preventable diseases. |  |  |  |
|  | Coughing of blood and chest pain while coughing are symptoms of symptom of vaccine preventable disease. |  |  |  |
|  | Dark urine and jaundice are also symptoms of vaccine preventable disease. |  |  |  |
|  | Vaccine preventable disease could be transmitted to a child from an infected person through airborne particles (droplet nuclei) as a result of coughing or sneezing. |  |  |  |
|  | Vaccine preventable disease could be transmitted through contaminated puncture wounds or contaminated sharp objects. |  |  |  |

# Section E: Attitude

**Instruction:** Kindly fill in the box by (√) the options that best represent your perspective for the statement/options as follow:

1. Strongly disagree
2. Disagree
3. Neutral
4. Agree
5. Strongly agree

| **Attitude** | | **Strongly disagree** | **Disagree** | **Neutral** | **Agree** | **Strongly agree** |
| --- | --- | --- | --- | --- | --- | --- |
|  | Vaccination is necessary for my child’s health. |  |  |  |  |  |
|  | I support vaccinations in children |  |  |  |  |  |
|  | I will recommend vaccination to others |  |  |  |  |  |
|  | I am not considering my child immunization as a waste of time |  |  |  |  |  |
|  | I have no doubt on childhood immunization. |  |  |  |  |  |
|  | A child should be immunized even if he/she is sick |  |  |  |  |  |
|  | Vaccination has no serious side effect |  |  |  |  |  |
|  | I would vaccinate my child even if there are mild side effects. |  |  |  |  |  |
|  | I would continue with my child immunization schedule even if my child develops any side effect. |  |  |  |  |  |
|  | I have no worries about vaccine safety |  |  |  |  |  |
|  | If I have a female child, I would vaccinate her because vaccine would not have negative impacts on her during child bearing age. |  |  |  |  |  |

# Section F: Outcome expectation

**Instruction:** Kindly fill in the box by (√) the options that best represent your perspective for the statement/options as follow:

1. Strongly disagree
2. Disagree
3. Neutral
4. Agree
5. Strongly agree

| **Outcome expectation** | | **Strongly disagree** | **Disagree** | **Neutral** | **Agree** | **Strongly agree** |
| --- | --- | --- | --- | --- | --- | --- |
|  | A child who is fully immunized will be protected against infectious diseases. |  |  |  |  |  |
|  | A child who receive Pentavalent vaccine will receive protection against 5 different infectious diseases. |  |  |  |  |  |
|  | Pentavalent vaccine gives protections against (hep b virus, diphtheria, pertussis, tetanus and haemophilus influenza-b infection). |  |  |  |  |  |
|  | A child who is not vaccinated is more likely to catch the infectious diseases (vaccine preventable diseases). |  |  |  |  |  |
|  | Children that are not vaccinated put others at risk. |  |  |  |  |  |
|  | Vaccine preventable diseases are very dangerous. |  |  |  |  |  |
|  | Infectious disease like VPDs can kill unvaccinated child. |  |  |  |  |  |

# Section G: Cultural beliefs

**Instruction:** Kindly fill in the box by (√) the options that best represent your perspective for the statement/options as follow:

1. Strongly disagree
2. Disagree
3. Neutral
4. Agree
5. Strongly agree

| **Cultural beliefs** | | **Strongly disagree** | **Disagree** | **Neutral** | **Agree** | **Strongly agree** |
| --- | --- | --- | --- | --- | --- | --- |
|  | I would vaccinate my child because it’s not against my culture. |  |  |  |  |  |
|  | My culture is in support of child vaccination |  |  |  |  |  |
|  | Childhood immunization is not a taboo in my culture |  |  |  |  |  |
|  | Our traditional leaders are encouraging us to comply with our child vaccination |  |  |  |  |  |
|  | In our culture, we believe childhood immunization is safe |  |  |  |  |  |
|  | In our culture, we believe childhood immunization enhance child immunity |  |  |  |  |  |
|  | In our culture, we believe childhood immunization provide protection to a child against certain vaccine preventable diseases |  |  |  |  |  |
|  | I will recommend my friend to vaccinate her child because it is not against our culture |  |  |  |  |  |

# Section H: Assumptions on Religious Regulations

**Instruction:** Kindly fill in the box by (√) the options that best represent your perspective for the statement/options as follow:

1. Strongly disagree
2. Disagree
3. Neutral
4. Agree
5. Strongly agree

| **Assumptions on Religious Regulations** | | **Strongly disagree** | **Disagree** | **Neutral** | **Agree** | **Strongly agree** |
| --- | --- | --- | --- | --- | --- | --- |
|  | I would vaccinate my child because it’s not against my religion. |  |  |  |  |  |
|  | My religion is in support of child vaccination |  |  |  |  |  |
|  | Childhood immunization is not a sin in my religion |  |  |  |  |  |
|  | Childhood immunization is halal in my religion |  |  |  |  |  |
|  | Our religion leaders are encouraging us to comply with our child vaccination |  |  |  |  |  |
|  | In our religion, we believe childhood immunization is safe |  |  |  |  |  |
|  | In our religion, we believe childhood immunization enhance child immunity |  |  |  |  |  |
|  | In our religion, we believe childhood immunization provide protection to a child against certain vaccine preventable disease |  |  |  |  |  |
|  | I will recommend my friend to vaccinate her child because it is not against our religion |  |  |  |  |  |

# Section I: Self-Efficacy

**Instruction:** Kindly fill in the box by (√) the options that best represent your perspective for the statement/options as follow:

1. Very hard
2. Hard
3. Easy
4. Very easy

| **SELF-EFFICACY** | | **Very hard** | **Hard** | **Easy** | **Very easy** |
| --- | --- | --- | --- | --- | --- |
|  | Right now, how easy or hard it would be for you |  |  |  |  |
|  | To come for your child immunization schedule. |  |  |  |  |
|  | To comply with your child immunization uptake even when the distance to hospital is long |  |  |  |  |
|  | To continue with your child immunization uptake even when the waiting time is long. |  |  |  |  |
|  | To comply with your child immunization even when the time is not convenient |  |  |  |  |
|  | To comply with your child immunization even when you are too busy |  |  |  |  |
|  | To continue with your child immunization uptake even when he/she develops a mild fever |  |  |  |  |
|  | To continue with your child immunization uptake even when he/she is having a pain at injection site. |  |  |  |  |
|  | To complete your child immunization uptake for Penta 3 |  |  |  |  |
