# Supplementary material for: Theory-based immunisation health education intervention in improving child immunisation uptake among antenatal mothers attending federal medical centre in Nigeria: A study protocol for a randomized controlled trial
Source: PLoS One. 2022 Dec 8;17(12):e0263436. doi: 10.1371/journal.pone.0263436 (PMC9731461; doi:10.1371/journal.pone.0263436)
Supplement: S6 File — (DOCX) [file pone.0263436.s007.docx]

**CHILD VACCINATION CARD**

**Note:** This patient is enrolling into a follow-up study, your help is needed to kindly fill the blanks provided on the card

**Serial Number: ………. Place of Delivery:**

**FMC Gusau**

**Others…**

| Antigens | Date given (DD-MM-YY) |
| --- | --- |
|  |  |
| BCG |  |
| Hep B-0 |  |
| OPV-0 |  |

Date of next vaccination………………

| Antigens | Date given |
| --- | --- |
|  |  |
| OPV-1 |  |
| Penta-1 |  |
| PCV-1 |  |

Date of next vaccination………………

| Antigens | Date given |
| --- | --- |
|  |  |
| OPV-2 |  |
| Penta-2 |  |
| PCV-2 |  |

Date of next vaccination………………

| Antigens | Date given |
| --- | --- |
|  |  |
| OPV-3 |  |
| Penta-3 |  |
| PCV-3 |  |
