# Supplementary material for: Theory-based immunisation health education intervention in improving child immunisation uptake among antenatal mothers attending federal medical centre in Nigeria: A study protocol for a randomized controlled trial
Source: PLoS One. 2022 Dec 8;17(12):e0263436. doi: 10.1371/journal.pone.0263436 (PMC9731461; doi:10.1371/journal.pone.0263436)
Supplement: Attachment — Submitted filename: Review comments Theory based intervention 16th August 2021.docx [file pone.0263436.s008.docx]

Review comments 16^th^ August 2021

A suggestion for title modification

A Study Protocol for a Randomized Controlled Trial to evaluate the effect of Theory-Based Immunization Health Education Among Antenatal Mothers Attending Federal Medical Center In Nigeria on Uptake of Child Immunization

Recommendation

An excellent protocol that is indeed likely to bring great improvements in an area in Nigeria where immunization if unacceptably low. It is informative and the authors are committed to sharing data on completion

References

Please add Wang et al - J Med Internet Res 2021 Feb 2;23(2):e21465. doi: 10.2196/21465.

The manuscript need some revision mainly of the grammar. Simple spell check will correct many of the errors included places where words are repeated.

Examples

Abstract

Sentence 1- Childhood immunization coverage is very low in Nigeria (31%) with Zamfara state being among the states with poorest coverage (<10%). Lack of maternal knowledge, attitude, outcome expectations, self-efficacy, cultural beliefs and assumptions of religious regulations of antenatal mothers towards childhood immunization contributed to poor childhood immunization uptake. This study aim is to develop, implement and evaluate the effects of an immunization health educational intervention with application of Social Cognitive Theory on pregnant women to improve knowledge, attitude, outcome expectations, self-efficacy, cultural beliefs and assumptions on religious regulations regarding childhood immunization uptake in Federal Medical Center Gusau, Zamfara State Nigeria.

Methodology

The study will be a double-blind parallel-group randomized controlled trial, where baseline data will be collected from 392 estimated antenatal mothers. After that they will be evenly randomized using randomly generated permuted block sizes (each containing two intervention and two control assignments). The study participants will be antenatal mothers of age 18 years and above who are in third trimesters attending Federal Medical Center Gusau, Zamfara state Nigeria during the study period and

fulfilled all the inclusion and exclusion criteria. The intervention group will undergo a five-health education session on immunization, which will be strictly guided by SCT based intervention module while the control group will receive usual care (standard care). Follow-up data will be collected using the same questionnaire at 6-weeks post-delivery, 10-weeks post-delivery and 14-weeks post-delivery. The generalized linear mixed model will be carried-out to determine the overall effect of the intervention after controlling for 14 potential confounding variables. An intention to treat analysis will also be carried-out. Childhood immunization uptake is the primary outcome while the secondary outcomes are: improved knowledge scores, attitude scores, outcomes expectation, self-efficacy scores, cultural beliefs scores and assumptions on religious regulations scores.

Discussion

The study will be a randomized controlled trial, that focuses on the effects of an Immunization health educational intervention with application of Social Cognitive Theory on pregnant women to improve knowledge, attitude, outcome expectations, self-efficacy, cultural beliefs and assumptions on religious regulations regarding childhood immunization uptake in Federal Medical Center Gusau, Zamfara State Nigeria.
