# Supplementary material for: Theory-based immunisation health education intervention in improving child immunisation uptake among antenatal mothers attending federal medical centre in Nigeria: A study protocol for a randomized controlled trial
Source: PLoS One. 2022 Dec 8;17(12):e0263436. doi: 10.1371/journal.pone.0263436 (PMC9731461; doi:10.1371/journal.pone.0263436)
Supplement: Attachment — Submitted filename: Pone Response to reviewers.docx [file pone.0263436.s009.docx]

Reviewer #1: The authors plan an important study. The manuscript submitted requires revision to fully illustrate the rationale for selecting the study design and the particular intervention as well as how it differs from the standard of care. Overall, the manuscript would benefit from more concise presentation of more detailed information, most especially about the methodology planned.
Specifically:
1/Background -
a/Too lengthy; reduce by about 1/3 in length.

Has been addressed (*kindly refer to page 3-7)*
b/Focus of the narrative drifts; concentrate on the immunization performance in the selected state, the reasons behind that, how the proposed intervention addresses the gaps and then state the research questions posed.

Has been addressed (*kindly refer to background section)*

2/Study design -
a/Explain how this design is the most appropriate to address the research questions.

Randomized controlled trial (RCT) study design is choosing in order to answer the research questions because it is regarded as the most rigorous and robust research method used for determining whether there is an existence of a real cause-effect relation between an intervention and an outcome in health research. Therefore, as we are planning of delivering health education intervention to antenatal mothers RCT will be the best study design to evaluate the effectiveness of our intervention. Moreover, high-quality evidence can be generated through conducting a randomized controlled trial when assessing the effectiveness and safety of an intervention and clinical practice decisions are based on evidence obtained from well-conducted RCTs when available. The main reason why evidence based on RCTs is regarded to be of the highest quality is that evidence based on observational study design is more prone to bias while in evidence based on RCTs, blinding and random allocation of participant to either intervention or control group reduce the likelihood for occurrence of bias.

b/Blinding - It is not possible to blind the participants to the assigned study group because they will know if they participate in the group intervention. Please rectify the description of blinding. The participants will be blinded against the participants’ groups. The participants wouldn’t be aware of which intervention they will be receiving until the trial is over.

c/Contamination; explain how women who have receive the intervention will be stopped from influencing immunization seeking behavior of the control women. Contamination will probably be one of the limitations of our study as the possibility of contamination from information sharing which could occur at home or other meeting places cannot also be excluded. However, mothers living in the same household will not be included in order to minimize possible contaminations.

3/Intervention -
a/Explain the development of the intervention and if it has been tested previously to convince the reader that it is appropriate and potentially feasible. One concern I have is the duration of the single session; in my experience, women in late pregnancy often are not comfortable to spend the whole day at the clinic and should be resting for some of the day.

For the development of the intervention, kindly refer to “development of the immunization health education intervention modules” under the immunization health education intervention modules section *(kindly refer to page 15*). The intervention has not been tested previously. However, we are in the process of conducting a piloting test as already planned.

For the duration, it has been amended.

b/Itemise the components of the intervention.

Has been attached as a separate document (kindly refer to S8 Table 2: component of the intervention (DOCX).

State whether the transport reimbursement and Pampers are considered elements of the intervention.

Has been addressed. (*Kindly refer to line 7 of intervention section page 12)*

c/Describe the standard of care in detail. Mention whether women assigned to the control arm will receive similar reimbursements and incentives as those in the intervention arm.

Has been addressed

For standard care *(kindly refer to line 6-10 of page 13)* and for reimbursements *(Kindly refer to line 5 of page 13 under intervention section).*

e/Pilot study - There is no explanation of the elements that will be assessed during the pilot stage and whether the women will also be in late pregnancy (35wks).

Has been addressed. *(Kindly refer to line 4-8 of quality control of the module content section page 17).*

4/Sample size – The study is powered to detect an effect size of about 20%. Explain whether this difference would lead to meaningful impact in the state immunization program once scaled up, if efficacious.

Yes, it will hopefully lead to meaningful impact as standard formular was used to obtained the study sample size. *(kindly refer to line 1-7 of page 10 under sample size determination section and Refer to Supporting document 2).*

Reviewer #2: The manuscript entitled ‘Theory-Based Immunization Health Education Intervention in Improving Child Immunization Uptake Among Antenatal Mothers Attending Federal Medical Center In
Nigeria: A Study Protocol for a Randomized Controlled Trial.

Comments
Study Design
The content in study Design (Page 9) and randomization (Page 10) is repeated.

Has been amended (*kindly refer to page 8 and 9).*

Random selection
Page 12, the information on who prepared and provided the sampling frame list and who performed the recruitment based on the table random numbers to be stated.

The sampling frame list will be prepared and provided by the hospital staff working in the Medical Records Department. Likewise, the recruitment based on the table random numbers will be performed by the hospital nurse from Antenatal Care Department. Both of them will not be participated in any of the study process. *(kindly refer to page 10 under random selection section)*.

Questionnaire quality control
Page 17, the statement ‘The questionnaire is first developed in English language which was later translated into Hausa language via the process of translation and adaptation of instruments figured by the World Health Organization [68]’ is repeated again in the Questionnaire Development section.

Has been amended. *(Kindly refer to page 17-18 under questionnaire development section and questionnaire quality control).*


Data Analyses
Proper citation for the statistical software to be used.

Descriptive Statistics
How sure mean and sd will be employed in the study and what happened if the data are skewed. *(Kindly refer to line 4-7 of page 18 under data analyses*).

Inferential statistics
1 or 2-tailed test for Fisher’s exact test and Independent t test to be stated. Why independent t test (assuming data are normally distributed) is used when pairwise comparison can be achieved in repeated measures.

Already stated under inferential statistics section.

For the statement ‘The estimated outputs that will be displayed will then be pooled into a single data set, after which the Generalized Linear Mixed Models (GLMM) analysis’ what estimated outputs and single data set refers to be clearly stated.

*Kindly refer to page 19 under data analyses section.*

Repeated measures one-way analysis of variance (ANOVA) to be written as one-way repeated measures ANOVA.
has been amended.

The actual name for the mixed design repeated measures ANOVA from the statistical software to be used.

*Has been addressed.*

It is not clear how these statistical tests (one-way repeated measures ANOVA, GLMM, mixed design repeated measures ANOVA will be employed in the analysis as these tests involved repeated measures. This needs to be clearly explained.

What is the reason one-way repeated measures ANOVA is employed when two groups are involved in the study?

*The reason why one-way repeated measures ANOVA is employed is to determine within-group differences with time for knowledge scores, attitude scores, outcome expectation scores, self-efficacy scores, cultural beliefs scores and assumptions on religious regulations scores.*

Also for the secondary outcomes, there are many outcomes studied. MANOVA approach could be considered e.g. repeated measures MANOVA etc provided the assumptions are fulfilled.

The subtitle normality test, descriptive statistics inferential statistics to be removed. All the information under these subtitles can be placed under Data Analyses or Statistical Analyses section.

*Has been amended. (Kindly refer to page 18-20 under data analyses section).*

Figure 1 requires cosmetic changes i.e arrows

Has been amended. *(Refer to page 15 under study process section).*

Some sections in the Materials and Methods require reorganization including statistical analyses and the manuscript requires grammar check and English editing.

*Has been addressed.*

Reviewer #3: Great work requiring proper spelling and language check. An excellent protocol that is indeed likely to bring great improvements in an area in Nigeria where immunization if unacceptably low. It is informative and the authors are committed to sharing data on completion

Several words are repeated of misspelt

*Has been addressed.*

Review comments 16^th^ August 2021

A suggestion for title modification

A Study Protocol for a Randomized Controlled Trial to evaluate the effect of Theory-Based Immunization Health Education Among Antenatal Mothers Attending Federal Medical Center In Nigeria on Uptake of Child Immunization

Has been addressed. *(kindly refer to line 1-3 of page 1).*

Recommendation

An excellent protocol that is indeed likely to bring great improvements in an area in Nigeria where immunization if unacceptably low. It is informative and the authors are committed to sharing data on completion

The manuscript need some revision mainly of the grammar. Simple spell check will correct many of the errors included places where words are repeated.

*Has been addressed entirely.*
