# Supplementary material for: Theory-based immunisation health education intervention in improving child immunisation uptake among antenatal mothers attending federal medical centre in Nigeria: A study protocol for a randomized controlled trial
Source: PLoS One. 2022 Dec 8;17(12):e0263436. doi: 10.1371/journal.pone.0263436 (PMC9731461; doi:10.1371/journal.pone.0263436)
Supplement: Attachment — Submitted filename: PONE Response letter 2022.docx [file pone.0263436.s010.docx]

1) The fluency of the written English varies throughout the manuscript. As PLoS ONE does not have any copy-editing services, this will need to be improved before the manuscript can be considered acceptable. *Has been addressed all over the manuscript*.

2) The question of "blinding" which Reviewer 1 raised is not a question of labelling. Yes, it is clear that assignment to intervention vs control can be hidden from the study team members who evaluate the outcomes. But it appears from the description of the intervention that it is so different from the control (local standard of care), that it will be obvious to the participants which group they are in. If the participants cannot be blinded (because the intervention is obviously different from the control) then keeping the evaluating team blinded would yield a single-blind study. This could be acceptable but should be described. *Kindly refer to page 12 line 1-2 under blinding section.*

3) Reviewer 1 also raised a potential confounding factor. As it appears from the description that participants in the intervention arm will be paid a lot more money than participants in the control arm, it possible that any differences in outcomes could merely be due to the financial incentives. Is there a way to pay the two groups of participants the same amount of money? *Kindly refer to page 13 line 4-7 under intervention section.*
